# Genotype Distribution of *Enterobius vermicularis* Isolates from Northwest Provinces of Iran

**DOI:** 10.4314/ejhs.v33i3.6

**Published:** 2023-05

**Authors:** Kaveh Figh Shilanabadi, Fatemeh Khadivi Derakhshan, Saber Raeghi

**Affiliations:** 1 Department of Biology, Urmia Branch, Islamic Azad University, Urmia, Iran; 2 Cellular and Molecular Research Center, Cellular and Molecular Medicine Research Institute, Urmia University of Medical Sciences, Urmia, Iran; 3 Department of Parasitology and Mycology, Faculty of Medicine, Urmia University of Medical Sciences, Urmia, Iran

**Keywords:** Enterobius vermicularis, cox1, Phylogenetic, Iran

## Abstract

**Background:**

Human infection with *Enterobius vermicularis* occurs worldwide. The most common clinical manifestation of a pinworm infection is an itchy anal region. This parasite is incidentally found in appendicitis. This study aims to characterize and genotype this parasite from different samples inferred by mt-DNA.

**Methods:**

Forty appendectomies for acute clinical appendicitis, 40 positive scotch-tape samples, and 10 adult females worm isolated from patients. Genetic differentiation, haplotype differences, and isolates population structure were analyzed based on the cytochrome c oxidase subunit I (*cox*1) gene.

**Results:**

It has been demonstrated that all isolations in the appendectomies specimens are similar, and the genetic difference divergence is seen in adult worm specimens. The neutral indices of the samples did not show a significant difference and show that there is no intra-specific and population distribution diversity.

**Conclusion:**

Our results show different haplotypes in the B type of E. vermicularis population and add new information about genotyping of these parasites in Iran. In comparison with other studies, intra-specific variation of this parasite from Iran was observed.

## Introduction

*Enterobius vermicularis* is a cosmopolitan distributed intestinal nematode in children that cause pinworm disease Oxyuriase. The transmission of this parasite is directly in the human population. The infection of this parasite is often asymptomatic. Other symptoms attributed to this parasite include itchy nose, appendicitis, vulvovaginitis, runny mouth, and gnashing of teeth at night with ectopic migration (1, 2).

Relevant studies identified different prevalence rates for infection of *E. vermicularis* (3). The prevalence of *E. vermicularis* in humans in different parts of Iran is 0.3% to 66.14% in various studies (3, 4, 5). In several countries, molecular and genotypic studies have been performed using mitochondrial markers (mt-DNA) to elucidate the genetic diversity of *E. vermicularis*, mainly to identify some patterns like types or specific haplotypes that may be associated with human isolates based on eggs and adult worms. Several phylogenetic studies from Asian and European countries have identified three populations or subtypes: A, B, and C (6, 7).

In addition, molecular studies of this *E. vermicularis* parasite previously performed in Iran have shown type B in humans. Genotypes A and B have been isolated from humans and animals like chimpanzees, but genotype C has only been reported in chimpanzees. However, no study reports genotype C of *E. vermicularis* in humans.

Molecular approaches and the genetic diversity indices help us find the adaptation of parasites. Considering lake of adequate information on the genotype of *E. vermicularis* in Iran, this study was aimed at characterization and genotyping this parasite from different samples inferred by mitochondrial DNA to get the parasitic gene flow among diverse populations and its relative comparison with other data in other studies.

## Materials and Methods

**Figure 1:**
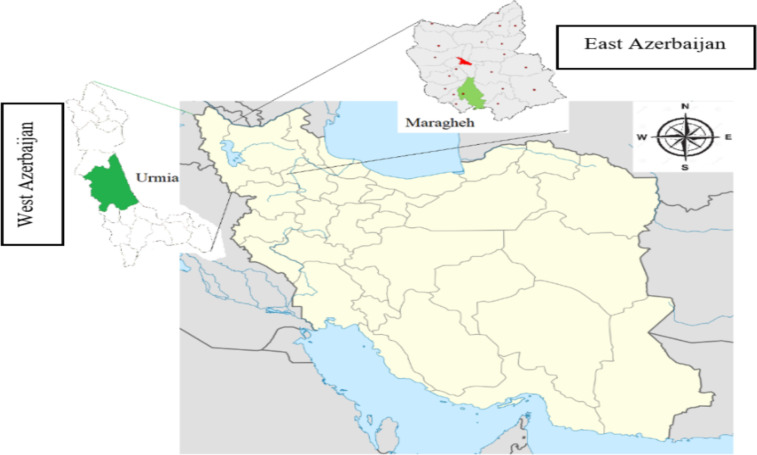
Study areas of E. vermicularis in the northwest of Iran (West & East Azerbaijan provinces).

**Study locations and samples collection**: Urmia city (37°32′55″N 45°04′03″E) is the center of West Azerbaijan province in Iran, and it is situated at an altitude of 4,360 ft above sea level. Also, Maragheh city (37°23′21″N 46°14′15″E) is located in the southwest of East Azarbaijan province on the south hillside of Sahand Mountain and situated at an altitude of 4,845 ft above sea level. The climate of the two cities is temperate, disposed to cold and humid (Figure 1). A total of 40 appendectomies for acute clinical appendicitis archived in laboratories that have been kept for 2-10 years and 40 positive scotch tape samples from clinical laboratories (20 Urmia, 20 Maragheh) were collected. 10 Adult female worms isolated from the studied regions were tested using a microscope and collected for DNA extraction.

**DNA extraction and sequencing**: According to the Ferrero study, for the isolation of eggs, pieces of tape were crushed, and eggs were isolated for DNA extraction (8, 9). Adult female worms isolated from the studied regions were tested using a microscope and used for controlling in DNA extraction and polymerase chain reaction (PCR) method. Microdissected appendices tissue distinguished as *E. vermicularis* causing acute appendicitis were used to extract DNA by xylene treatment, which dissolves the paraffin from the tissue and then rehydrated using a series of ethanol washes (10). According to kit instructions, DNA of obtained tissues was extracted with (ambio®, Sambio™, Iran). Total DNA from each sample was extracted with a High Pure PCR Template Preparation Kit (Dynabio^®^, Takapouzist, Iran), according to the instructions, and all of which were stored at - 20 °C until use. The *cox1* gene was amplified using primers based on Piperaki et al. study (11).

DNA fragments of target region were analyzed using the PCR reaction with a pair primer forward (EVM1: 5 - TTTTTGGTCATCCTGAGGTTTATATTC-3) and reverse primers (EVM2: 5-CACATTATCCAAAATAGGATTAGCC-3).

The total volume of the reaction was 30 µl containing 3 µl DNA template, 10 µl distilled water, 10 pmol of each primer, and 15 µl master mix (amplicon).

Reaction cycles consisted of an initial denaturing step at 94°C for 5 min, followed by 35 cycles at 94°C for 90 s, 57°C for 60 s, and 72°C for 45 s, with a final extension at 72°C for 10 min using a gradient thermocycler. DNA fragments were analyzed by 1.5% agarose gel electrophoresis and sequenced by Bioneer Company using the same primers.

**Phylogenetic analysis**: Sequences have been aligned and unlike the sequences from the region. In contrast to existing sequences from the area associated with E. *vermicularis* available in the GenBank, using Chromas 2.2 and multiple, some alignment was done with the data linked to *E. vermicularis* from Iran deposited in GenBank. Phylogenic analyses based on *cox1* sequence data were conducted to the fullest possible using MEGAX (12). All sequences were run unordered and equally weighted. Alignment gaps were treated as missing data, and bootstrap analyses were done using 1000 replicates.

**Genetic diversity indices**: The number of segregating sites, diversity indices (Haplotype diversity: Hd and Nucleotide diversity: π), and neutrality values (Tajima's *D* and Fu's Fs tests) were calculated by DnaSP software version 5.10(13). The degree of gene flow among the *E. vermicularis* populations was evaluated using a pairwise fixation index (*F*st: F-statistics) (14, 15).

## Results

**Figure 2:**
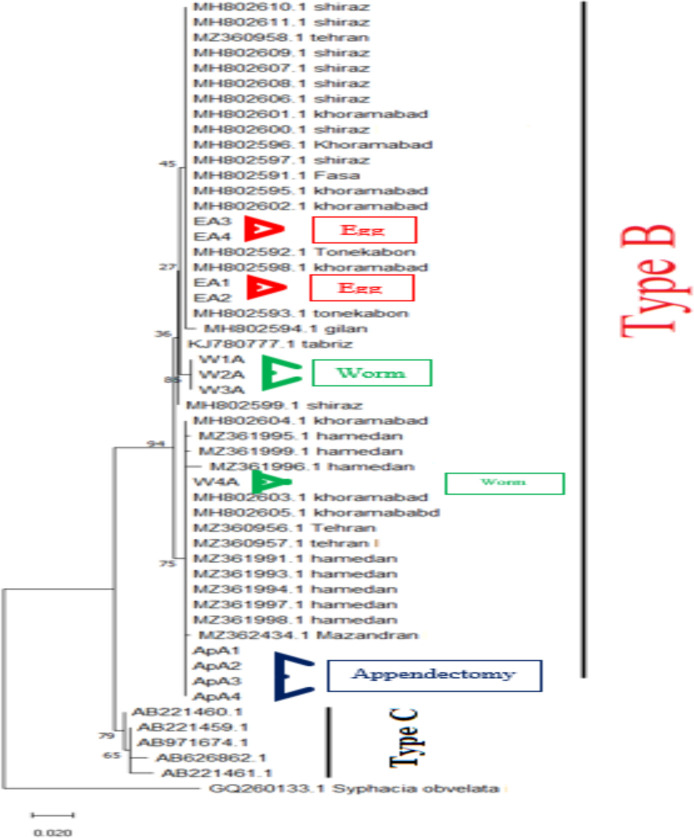
Phylogenetic relationship based on cytochrome C oxidase subunit 1 (cox1) gene sequences of E. vermicularis in this study next to type B. The tree was conducted by Maximum likelihood using MEGAX with Syphacia obvelata designated as an outgroup. Scale bars indicated nucleotide substitutions per site.

An approximately 390 bp band was amplified from 22 appendectomy samples and 35 egg isolates from tapes and ten adult worms of *E. vermicularis* as positive control samples. Sequence analysis was performed for 24 PCR products consisting of appendectomy, egg isolates from tapes, and adult worms (8 from each group) to characterize the genotype of *E. vermicularis*. Phylogenic analyses of *E. vermicularis* haplotypes based *on the cox1* gene and type of nucleotides in Iran were conducted by Maximum likelihood (ML) using MEGAX with *Syphacia obvelata* designated as an out-group shown in Figure 2. The sequences obtained in this study were registered in GenBank under the following accession numbers: OL774836 to OL774839 and OL773357-58 and OL773361-62. The percentage of similarity and difference in genetic sequences in all three types of samples is shown in Table 1. It has been demonstrated that all isolations in the appendectomies specimens are similar, and the genetic difference divergence is seen in adult worm specimens.

**Table 1:** Percent Identity and Divergence of *E. vermicularis* from 3 types of samples in this study

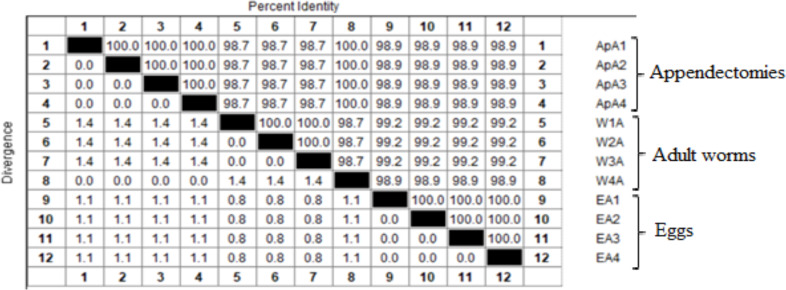

Using DnaSP 5.10 software, haplotype differences (Hd), nucleotide differences (π = Nd), and neutrality indices of the samples studied in this study and recorded from different parts of Iran in GeneBank were calculated (Table 2).

**Table 2:** Haplotype differences of *E. vermicularis* population in this study and Iran based on *cox1* gene sequence

Population	Shiraz	Khoramabad	Tehran	Gilan
Azerbaijan (this study)	0.34524	0.00000	0.03392	0.00000
Shiraz	-	0.14054	0.57391	0.07143
Khoramabad	-	-	0.10323	0.00000
Tehran	-	-	-	0.10145

N: number of sequences, Nh: number of haplotypes, Hd: haplotype diversity, S: segregation sites, Nd (π): nucleotide diversity.

The results show that the haplotype diversity in the samples of Azerbaijan is 0.712, and the number of haplotypes in this region is 3. On the other hand, the neutral indices of the samples of Azerbaijan did not show a significant difference and show that there is no intra-specific and population distribution diversity in the samples of Azerbaijan (this study).

Fst values between various populations of *E. vermicularis* were calculated by the Dnasp5 software package with the nucleotide data set of *cox1* gene. The results showed that there was the slightest difference in the genetic structure of the population in our studied samples, such as Azerbaijan, Khorramabad, and Gilan north of Iran (Fst = 0), and the highest genetic population distance of Azerbaijan with the samples of Shiraz in the southwest of Iran (Fst = 0.34524). This index (Table 3).

**Table 3:** The degree of gene flow (Fst) among populations of *E. vermicularis* compared with the studied samples

Population	Shiraz	Khoramabad	Tehran	Gilan
Azerbaijan (this study)	0.34524	0.00000	0.03392	0.00000
Shiraz	-	0.14054	0.57391	0.07143
Khoramabad	-	-	0.10323	0.00000
Tehran	-	-	-	0.10145

## Discussion

Genotypic studies of worms have multiplied recently and provided valuable information on geographical distribution. Molecular studies from genetics and population allow us to recreate the evolutionary relationships between parasites and hosts and understand their geographical distribution, diversity, and the etiology of the disease (16, 17, 18).

Based on mitochondrial DNA markers, three types of A, B, and C were identified from the *E. vermicularis* (19, 20, 21). Although this variety is in three groups in different parts of the world, type C is only seen in animals, and type B is dedicated to human specimens (22).

The *cox*1 tree topologies indicated that all samples were typed B. These results are similar to Tavan et al. from Shiraz and Khoramabad in 2020 (23). It seems that genotype B is the only type of *E. vermicularis* in Iran. However, a study from Iran showed that the B type is divided into two branches (24), but our results indicate different haplotypes in the type B samples in Iran, and there are intraspecific variations in this type. Diversity indices in *E. vermicularis* population from 4 other locations in Iran show the variations of haplotypes. The findings could be attributed to high mutation rate in cox1 gene. Also, Tavan (2020) shows the genetic variability in the studied *E. vermicularis* population (23). A study conducted by Piperaki et al. in 2011 showed that the single haplotypes in the studied human population were probably related to the principal and common source of infection (11). Like our study, they could not find any clear association between the haplotypes and the origin or location studied.

Neutrality indices of the isolates from appendectomies did not show significantly different intra-specific diversities. In other words, despite the haplotype diversity in Iranian samples, these changes did not lead to intra-specific diversity.

Balloux describes genetic discrimination grade from infra-population in 2002 (25, 26). *F*_ST_ will seriously underestimate differentiation in highly structured populations. Fst values between the *E. vermicularis* population of this study and Gilan and Khorramabad are 0, indicating low genetic differentiation in or same population. This finding could be attributed to little direct movement or interaction of infected human populations. The limitation of this study was that the number of sequences was related to isolates for more comprehensive details to compare the sequences with each other.

In conclusion, our samples show different haplotypes in the B type of *E. vermicularis* population and add new information about genotyping of these parasites in Iran. In comparison with other studies, intra-specific variation of this parasite from Iran was observed.
